# Photoperiod and Temperature as Seasonal Cues for the Initiation of Brood Rearing in Honeybees

**DOI:** 10.1002/ece3.72066

**Published:** 2025-08-27

**Authors:** Zeynep N. Ulgezen, Coby van Dooremalen, Frank van Langevelde

**Affiliations:** ^1^ Wildlife Ecology and Conservation Group, Department of Environmental Sciences Wageningen University & Research Wageningen the Netherlands; ^2^ Wageningen Plant Research Wageningen University & Research Wageningen the Netherlands

**Keywords:** *Apis mellifera*, brood‐rearing activity, climate change, phenology, plant–pollinator interaction, seasonal timing

## Abstract

The timing of seasonal life cycle events in many organisms is regulated by environmental cues, and understanding these relationships is essential for predicting species' responses to climate change. In honeybee colonies, brood rearing must align with floral resource availability to ensure colony growth and survival. However, the cues that initiate brood rearing remain unclear. While temperature is hypothesized to be influential, the role of photoperiod is not well understood. We used field experiment data in 2021 and 2022 from 95 colonies across eight European countries to examine how temperature and photoperiod interact to regulate the initiation of brood rearing in early spring. We found that both cues are important: longer day lengths and higher temperatures were associated with a higher probability of brood rearing, with temperature having a stronger effect under shorter day lengths and diminishing as day length increased. Notably, the strong positive effect of photoperiod suggests it may play a more prominent role than previously recognized. This interaction underscores the complexity of phenological regulation in honeybees. As global temperatures rise and seasonal patterns shift, colonies may struggle to synchronize brood rearing with floral resources. Our findings suggest that incorporating multiple environmental cues and species‐specific sensitivities may improve predictions of climate‐driven shifts in plant–pollinator interactions.

## Graphical abstract

We examined how temperature and photoperiod interact to regulate the initiation of brood rearing in honeybee colonies across multiple European sites. Both cues were important, with photoperiod playing a stronger role than previously recognized. These findings suggest that multiple environmental cues may need to be considered when predicting pollinator responses to climate change.

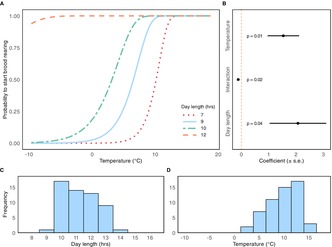

## 1 Introduction

Understanding phenological cues is crucial in ecology, as they govern life cycle events in both plants and animals. For pollinators, these cues play an important role in the timing of collecting food resources, facilitating pollination, a process that supports global food production (Gallai et al. 2009) and biodiversity (Wei et al. 2021). Climate change may alter the timing of key life cycle events like plant flowering and pollinator emergence, which may lead to mismatches between species (Hegland et al. 2009). Such phenological decoupling can jeopardize both pollinator populations and plant reproduction (de Manincor et al. 2023), threatening ecosystem stability. These potential impacts highlight the need to understand the phenological cues that drive pollinator responses.

Honeybees ( *Apis mellifera* ) are one of the most effective (Rader et al. 2009) and important pollinator species (Hung et al. 2018). There have been high losses in colonies over the past several decades due to a multitude of interacting stressors including parasites and pathogens, as well as environmental factors such as lack of floral resources and climate change (Goulson et al. 2015; Potts et al. 2010; Sánchez‐Bayo and Wyckhuys 2019). Honeybee colonies in temperate climates show adaptations to changing environmental conditions, such as seasonal variation in temperature and resource availability, that may become stressful when they challenge colony resilience (Ulgezen et al. 2021). In early winter, when resources are scarce or absent, colonies rely on stored honey and cease brood rearing to conserve energy, forming a dense cluster to minimize heat loss (Seeley and Visscher 1985; Southwick 1985). Honeybees then actively thermoregulate within this cluster, maintaining a stable core cluster temperature (Southwick 1985). When environmental conditions improve, around late winter or early spring, colonies reinitiate brood rearing, increasing the cluster temperature to create optimal conditions for larval development (Fahrenholz et al. 1989). During this period, colonies can rely on stored pollen to meet the nutritional demands of brood rearing, but shortages of pollen, especially in poor spring conditions, can limit or interrupt brood production (Mattila and Otis 2006; Seeley and Visscher 1985). In contrast, in (sub)tropical regions where winters are absent, but wet‐dry seasons can influence floral availability, colonies can forage and rear brood year‐round, with less pronounced seasonal changes (Schneider and McNally 1992). Although colonies are adaptable to environmental changes and seasonal differences, high climate variability can lead to colony mortality (Switanek et al. 2017), by possibly exceeding the colony's adaptive capacity. Given that future global climate changes, alongside ongoing land use modifications such as habitat loss and agricultural intensification, are likely to worsen unfavorable conditions by increasing extreme weather events and reducing floral availability and diversity, honeybee populations may face heightened challenges. Understanding honeybee phenological cues that drive seasonal brood rearing is therefore important to predict and mitigate impacts on colony survival.

The timing of brood‐rearing initiation is critical, as it determines workforce availability when floral resources emerge. Improper timing, either too early or too late, potentially causes colony‐level problems (Seeley and Visscher 1985). Initiating brood rearing too early may deplete the colony's limited winter food stores, risking starvation before flowers bloom, especially if there is asynchrony between food resources and honeybees due to differing phenological cues. Low resource availability, such as reduced pollen, can hamper colony growth and reproduction (Requier et al. 2017; Schmickl and Crailsheim 2001; Ulgezen et al. 2024). Similarly, limited resources in spring have been found to reduce colony size (Farrar 1936) which is important for reproduction (Smith et al. 2014) and spring survival (Harbo 1986). It has also been shown that early onset of brood rearing can increase the colony's vulnerability to the brood parasite of *Varroa destructor* (Nürnberger et al. 2019), as it allows for more reproductive cycles of the mite and increases mite population growth. Shifts in brood phenology may further complicate *V. destructor* management, as treatments must be carefully timed to avoid harming brood (O'Connell et al. 2025). This parasite is considered to be one of the most significant threats to honeybee colonies, and if unmanaged, it can often lead to colony decline or collapse (Rosenkranz et al. 2010).

Delaying the timing of brood initiation can also have consequences, such as postponing workforce development and reducing the colony's ability to exploit spring bloom, which is important for replenishing food stores and growth. Colonies that start brood rearing later have smaller colony sizes and lower amounts of stored honey during the season (Nürnberger et al. 2019). Proper timing also ensures that the colony is ready for key life cycle events, such as swarming, the colony's reproduction. Delayed onset of brood rearing can cause colonies to have later swarms, which are more likely to starve in winter (Seeley and Visscher 1985). Therefore, the timing of brood rearing must align with environmental conditions to maximize resource acquisition, colony growth, and survival.

The initiation of brood rearing in honeybees is thought to be influenced by multiple environmental cues, particularly photoperiod and temperature, though the exact drivers remain unclear (Nürnberger et al. 2018; Ulgezen et al. 2024; Villagomez et al. 2021). Temperature, a cue for many insect species, can trigger earlier emergence and increased activity during warmer winters and springs in temperate regions (Forrest 2016). Long‐term studies looking at emergence trends show that bee species appear earlier with rising spring temperatures (Cane 2021; Wyver et al. 2023), including honeybees (Gordo and Sanz 2005), highlighting its potential significance. Yet, further studies in honeybees show varying results. While temperature is hypothesized to intensify brood rearing activity, increasing the likelihood and duration of brood presence, it remains unclear whether it is an actual trigger for the onset of brood rearing (Nürnberger et al. 2018; Villagomez et al. 2021). Photoperiod (day length), though extensively studied for its role in regulating insect diapause, plays a more complex role (Forrest 2016). This complexity also applies in honeybees, where its function is even more equivocal compared with temperature. Earlier research suggests that longer day lengths increase brood‐rearing activity (Kefuss 1978). However, more recent research suggests that photoperiod does not influence the initiation of brood rearing nor brood‐rearing activity (Villagomez et al. 2021), but may modulate the response to temperature (Nürnberger et al. 2018). This unclarity in the role of environmental cues underscores the necessity for further research into the subject. Notably, much of this evidence specific to honeybees comes from controlled environment studies, with field experiments exploring how these cues interact under natural conditions being scarce.

Here, we investigated the roles of photoperiod and temperature in brood‐rearing initiation of honeybee colonies, by using data from field experiments conducted in eight European countries over 2 years. Field studies are particularly relevant, as they might offer insights into how colonies respond to different environmental cues under natural conditions. By understanding the influence of these environmental cues, we aim to provide a better understanding for predicting how shifts in phenological cues due to climate change could influence honeybee colonies and their ability to synchronize with flowering plants.

## 2 Methods

### 2.1 Experiment Set‐Up

**FIGURE 1:**
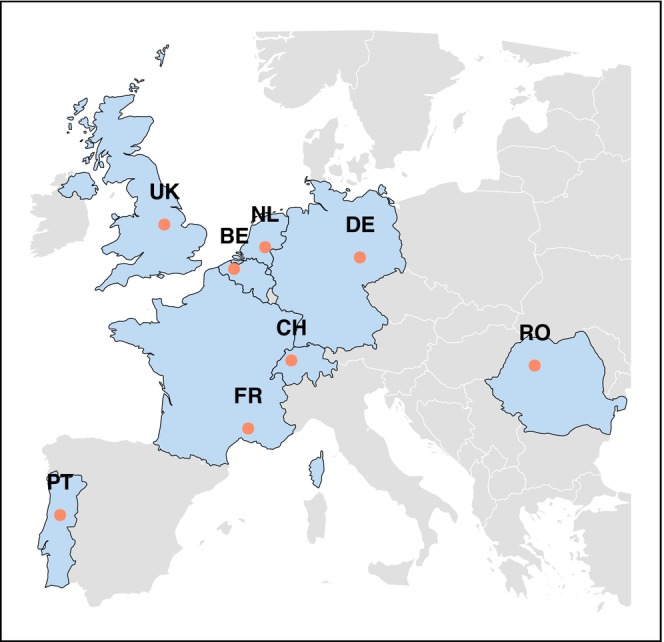
Geographic distribution of apiaries across Europe over different countries. Labeled points represent apiary locations (BE Belgium, CH Switzerland, DE Germany, FR France, UK United Kingdom, NL The Netherlands, PT Portugal, RO Romania).

All data used here were collected as a part of the EU Horizon 2020 B‐GOOD project (van Dooremalen et al. 2024). The fieldwork spanned over 3 years, starting from January 2020 to December 2022. For the project, apiaries were installed in eight different countries across Europe. These were: Belgium (BE), the Netherlands (NL), France (FR), Germany (DE), Romania (RO), Portugal (PT), United Kingdom (UK), and Switzerland (CH) (Figure 1). Each country had one apiary, and in each apiary, eight honeybee *(Apis mellifera)* colonies were set up. Colonies were established using local honeybee stocks from each country, typically representative of the region's ecotype and/or subspecies. The apiaries were installed and managed by research institutes from each country. Detailed information about the project setup, protocols, and methods can be found in van Dooremalen et al. (2024).

At the start of the experiments, all colonies were presumably healthy (no visible signs of disease, with an egg‐laying queen and with sufficient number of adult worker honeybees). The primary objective was to keep healthy colonies and provide care accordingly, including practices such as treatment against parasites and supplementary sugar feeding. These practices were adapted to local environmental and seasonal conditions. If a colony died at any point, when possible, it was replaced by a new colony. The type of hive and management practices represented the country standard for each apiary. Hence, instead of standardized protocols, guidelines were provided for specific beekeeping practices. More detailed information on colony management plans can be found in van Dooremalen et al. (2024). Information on apiaries, colonies, hives, management actions, and inspections was all recorded in the BEEP app (https://beep.nl/index.php/beep‐app), a web app specifically designed to track beekeeping actions.

Each colony was equipped with the BEEPbase Sensor System (https://beep.nl/index.php/measurement‐system‐2), which included a sensor for measuring in‐hive temperature (1‐Wire, DS18b20). The temperature sensor was placed at the center of the hive, between frames of honeybees, about 9 cm deep from the top of the frame. Measurements were recorded in 15‐min intervals, and data were transmitted from a long‐range (LoRa) gateway and recorded in the BEEP app. As the sensors were set up in spring/summer of 2020, in our analysis, we only used data from 2 years, 2021 and 2022, to capture the start of brood rearing.

### 2.2 Honeybee Brood Rearing

To investigate the start of brood rearing and brood‐rearing activity, we used the daily mean in‐hive temperature. The mean was calculated by taking the average of measurements between 00:00 and 02:000, to avoid any possible effects of management activities, and ensure that all honeybees were in the colony at the time of measurement. In the absence of brood, colony temperature is around 21°C (Fahrenholz et al. 1989). When brood rearing starts, honeybees maintain constant colony temperatures between 33°C and 36°C (Jones and Oldroyd 2006). This thermal stability is critical to healthy brood development, as brood reared at suboptimal temperatures may show behavioral impairments as adults (Becher et al. 2009; Tautz et al. 2003), and lower probability of survival (Wang et al. 2016). Hence, the initiation of brood rearing was recorded as the first day of the year when in‐hive temperature was 33°C or above, reflecting the lower bound of the optimal colony temperature range. Colony temperatures are typically stable and fluctuate only 1°C within a day (Seeley 1995). A colony was only considered brood rearing if 33°C was maintained for at least three consecutive days (Villagomez et al. 2021) to avoid false‐positives from brief temperature fluctuations unrelated to brood presence. If this was not the case, the next moment when these conditions were met was recorded as the first day of brood rearing. As the distance between the temperature sensor and the brood cluster may influence sensor recordings and lead to potentially erroneous and/or lagged recognition of the initiation of brood rearing, we used two additional methods to confirm the start day. First, we used the data from in‐hive temperature sensors to generate time series graphs. For each colony, we checked the first day in the leap in in‐hive temperature as a representation of shifting from a broodless state to the brood‐rearing state (Ulgezen et al. 2024). Second, throughout the B‐GOOD project, in winter once a month and from spring to autumn every 21 days, the presence of brood was recorded, including the time when brood was first observed. This was done by opening and visually inspecting the hives. We confirmed the start of brood rearing by comparing the start day from these different methods (Figure S1). After the initial moment of brood rearing, a colony was considered brood rearing when in‐hive temperature was above 33°C.

### 2.3 Temperature and Photoperiod

To explore the influence of ambient temperature on brood rearing, we used data from the global atmospheric reanalysis produced by the European Center for Medium Range Weather Forecasts (ECMWF; ERA5). The ERA5 dataset provides global climate and weather estimates from 1940 to present, with hourly temporal resolution and 0.25° × 0.25° spatial resolution (Muñoz‐Sabater et al. 2021). From this dataset, we obtained the mean daily temperature (air temperature at 2 m above surface) for each apiary location for 2021 and 2022. This parameter is in units of Kelvin, so it was converted to degrees Celsius (°C).

**FIGURE 2:**
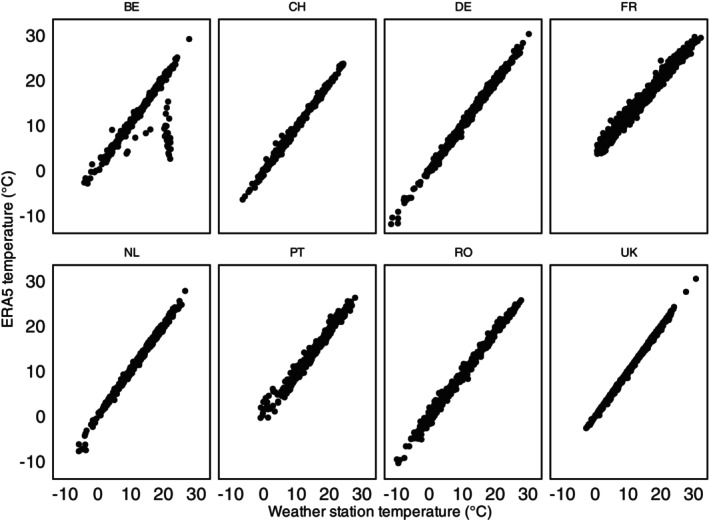
Correlation between ambient temperature data from local weather station and ERA5 per location (BE Belgium, CH Switzerland, DE Germany, FR France, UK United Kingdom, NL The Netherlands, PT Portugal, RO Romania).

Data on local weather, including temperature, was also collected at each apiary. Due to network connectivity issues, there was some loss of data, especially during winter and early spring. Therefore, we could not use these data for the analysis of the start of brood rearing. However, we compared the mean daily ambient temperature from apiaries to the data from ERA5 to confirm that it represented local conditions, using Pearson Correlation tests (Table S1). For all countries, the weather data from apiaries was highly correlated with the data from ERA5 (Figure 2), supporting the reliability of the data. There were slight discrepancies in temperature readings between the two data sources in Belgium (BE). While we do not know the exact cause, it is most likely due to erroneous temperature recordings by the local weather station. We noticed that the sensor had the same temperature reading for several days.

To investigate the effects of photoperiod on brood rearing, we calculated the total day length per day for each location using the *R* package “chillR” (Luedeling 2019), which estimates photoperiod based on calculations of sunrise and sunset times, using latitude and date as inputs.

### 2.4 Statistical Analysis

To examine the effect of ambient temperature and photoperiod on the start of brood rearing, we used a mixed‐effects Cox Proportional Hazards Model (coxme R package, Therneau 2015) with time‐varying covariates (Therneau et al. 2017). The focus here was on the first occurrence of brood. For each colony, every day they did not have brood was recorded as “0.” The first day when colonies started to rear brood was recorded as “1” (*n* = 58). For colonies that failed to start brood rearing or died during winter, data were entered as “0” (*n* = 37) on the last day that data were present. If this date was after spring, the last day was recorded as the 31st of May, since the latest brood initiation in our dataset occurred in April, and we presumed that if brood did not initiate by then, it would not start afterward. Colonies with too few consecutive data points or missing data to reliably determine the first day of brood rearing, due to connection issues or faulty temperature sensors, were excluded from the dataset to ensure accurate identification of brood initiation. The number of colonies per country included in the final analysis is shown in Table 1. The explanatory variables used in the model were photoperiod (day length), ambient temperature, and their interaction, with colony nested within country added as random effects. This random structure accounts for the environmental and management similarities within countries and colony‐level variation.

**TABLE 1:** Number of colonies per country used in the analysis of initiation of brood rearing and brood‐rearing activity.

Country	Colony
Belgium (BE)	13
Switzerland (CH)	11
Germany (DE)	13
France (FR)	11
United Kingdom (UK)	9
The Netherlands (NL)	14
Portugal (PT)	11
Romania (RO)	13

For the temperature variable, we used a 7‐day rolling mean of each day to account for the possible lagged or gradual effect of temperature on brood‐rearing initiation. This window was selected based on model fit: shorter windows decreased model fit (based on AIC), including using 1 day (i.e., instantaneous effect), while longer windows reduced temperature variation (Table S2). The 7‐day period balanced capturing a meaningful effect while preserving variation in temperature data.

To further explore brood‐rearing patterns, we assessed the average day of brood initiation per country and compared the corresponding ambient temperature and day length across countries. Separate Kruskal–Wallis tests were conducted for ambient temperature and day length to compare countries. Post hoc comparisons were performed using Dunn's Test.

We also investigated how photoperiod and ambient temperature influenced overall brood‐rearing activity, defined here as the presence or absence of brood, throughout spring. Specifically, we examined if these factors affected the probability of brood presence, regardless of when brood rearing started. For this, we used the days in which brood was present, marked as “1,” and absent, marked as “0,” until the end of spring (i.e., 31st of May). Analysis for brood presence was done by using a generalized linear mixed model (GLMM) with a binomial distribution and logit link function (lme4 R package, Bates 2011). Ambient temperature (daily mean instead of the smoothed 7‐day rolling mean), photoperiod (day length), and their interaction were included as explanatory variables, and colony nested within country was added into the model as random effects to account for colony‐level variation and clustering effects within countries. Data were scaled to compare effect sizes of predictor variables. The interaction term was retained in models only when it was statistically significant.

All statistical analyses were done using the software R version 4.3.1.

## 3 Results

### 3.1 Initiation of Brood Rearing

**FIGURE 3:**
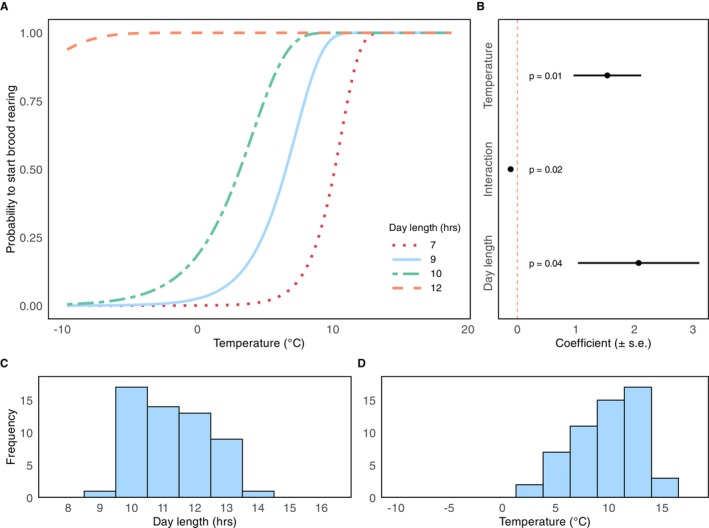
The influence of environmental cues on the initiation of brood rearing. (A) The predicted probabilities of brood initiation based on ambient temperature and day length, estimated using the mixed‐effect Cox model. (B) Estimates from the mixed‐effect Cox model for fixed factors and their significance levels. (C) Histogram of day length on the day colonies started brood rearing. (D) Histogram of ambient temperature on the day colonies started brood rearing. The y‐axis for histograms displays the entire range of values from the dataset. Ambient temperature values were calculated as the 7‐day rolling mean.

Both photoperiod and ambient temperature were strongly correlated to the start of brood rearing in honeybee colonies (Cox Model: X^2^ = 38, df = 5, *p* < 0.001). Longer day lengths and higher ambient temperatures were associated with a higher probability of the start of brood rearing (Figure 3A). Day length had a larger effect on brood rearing compared with ambient temperature (Figure 3B). There was also a negative interaction between the two factors, where the individual effect of each variable slightly reduced compared with when only one of them is high (Figure 3A,B). Our results suggest that the effect of temperature on brood‐rearing initiation is larger during shorter days, whereas in longer days photoperiod becomes more influential, lessening the direct impact of temperature. For example, the probability of starting brood rearing at 10°C was around 25% when day length was 7 h, but once day length reached 9 h or more, there was nearly a 100% probability of initiating brood rearing, regardless of further increases in ambient temperature. Most colonies started brood rearing when day length was around 10–12 h, and ambient temperature was around 10°C (Figure 3C,D).

**FIGURE 4:**
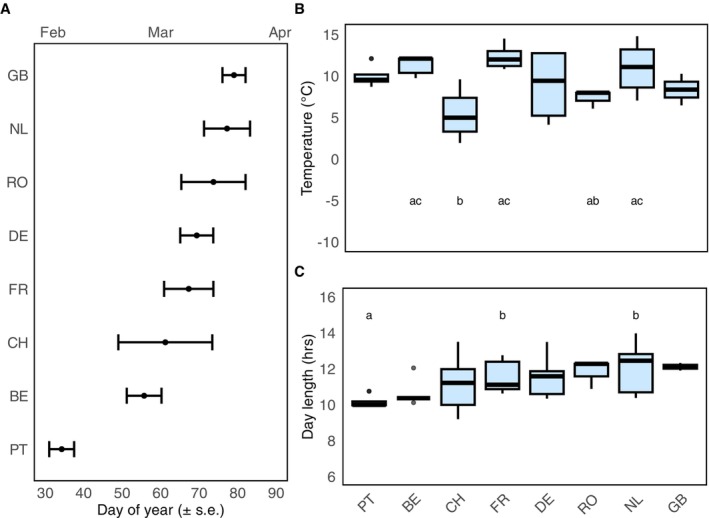
Country averages for the start of brood rearing (A) Mean day (± SE) of the year when colonies start to rear brood per country. Day 1 represents January 1st. (B) Median temperature when colonies start brood rearing per country. (C) Median day length when colonies start brood rearing per country. The *y*‐axis for day length and temperature displays the entire range of values from the dataset. Letters indicate significant differences between countries (Dunn's Test). No letters mean no differences were found.

Brood rearing in the colonies was initiated between February and March, with most colonies starting in March (Figure 4A). There were some differences between countries in the average ambient temperature (Kruskal–Wallis: *H* = 21, df = 7, *p* < 0.01) and average day length (Kruskal–Wallis: *H* = 28, df = 7, *p* < 0.001) at the time when brood rearing began (Figure 4B,C). We also examined whether brood initiation followed any latitude trends across countries, but found no clear pattern (Figure S2).

### 3.2 Brood‐Rearing Activity

**FIGURE 5:**
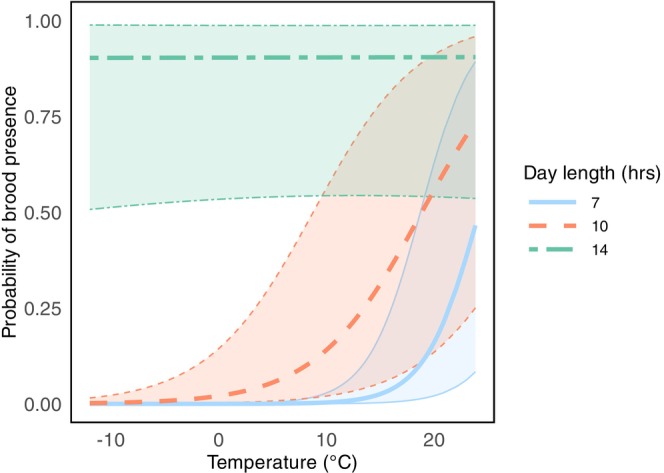
Probability of brood presence based on ambient temperature and day length. Shaded areas within lines represent 95% CI. Marginal *R* ^2^/conditional *R* ^2^ = 0.67/0.84.

Ambient temperature and photoperiod had a very similar effect on brood‐rearing activity (Figure 5). An increase in both ambient temperature and day length increased the probability of brood presence, with day length (GLMM: β = 3.16, s.e. = 0.09, *p* < 0.001) having a larger effect compared with temperature (GLMM: β = 0.87, SE 0.07, *p* < 0.001). We also found an interaction effect between ambient temperature and day length, and that as day length became longer, the effect of temperature on brood presence reduced (GLMM: temperature × day length, β = −0.90, SE = 0.08, *p* < 0.001). Comparable to the initiation of brood rearing, our results imply that these environmental predictors do not add up in their effects, but instead moderate each other.

## 4 Discussion

In this study, we investigated the effect of photoperiod (day length) and temperature as phenological cues for the initiation of brood rearing in honeybee colonies in early spring. We show that, for the first time under natural conditions, both of these factors serve as environmental cues. The interaction between these factors demonstrates a nuanced relationship, where temperature effects are larger during shorter days, and during longer days, this effect is reduced. We also found that brood‐rearing activity throughout spring followed a similar pattern, with both photoperiod and temperature positively influencing the probability of brood presence, including the same interaction effect. Our findings show that multiple environmental cues regulate seasonal timing and underline the complexity of phenological regulation in honeybee colonies. The use of field data across diverse European countries provides a robust understanding of how honeybee colonies respond to changing environmental cues in temperate regions.

Temperature has been recognized as a cue for the initiation of brood rearing in honeybee colonies, and our findings here are similar to earlier studies suggesting that warmer temperatures are associated with earlier and more active brood rearing (Nürnberger et al. 2018; Villagomez et al. 2021). Regarding photoperiod's role, previous findings have been ambiguous: some studies suggest an influence on brood‐rearing activity (Kefuss 1978), others find no effect (Villagomez et al. 2021), while some indicate a modulatory role (Nürnberger et al. 2018). Our findings align with the latter, where we found a modulatory interaction between photoperiod and temperature. However, we also observed a strong positive influence of photoperiod. Increased light intensity, along with temperature, has been shown to trigger food consumption in overwintering colonies, suggesting that light plays a role in honeybee activity during winter (Norrström et al. 2021). This influence of light may explain why we observed a strong effect of photoperiod.

A possible explanation for the discrepancy in the effect of photoperiod with previous studies could lie in the differences in experimental conditions. Many of the previous studies were conducted in controlled climate chambers, where environmental factors are strictly regulated. This may diminish or obscure the effects of photoperiod. Studies in chronobiology show that animals can respond very differently to light and temperature in lab versus natural settings, possibly due to the involvement of other environmental conditions or richer stimuli (Gattermann et al. 2008; Vanin et al. 2012). Similarly, genetic markers in plants linked to photoperiod response in controlled conditions can be undetectable in the field (Weinig et al. 2002), underscoring the potential differences. Furthermore, discrepancies between controlled and natural environments regarding phenological events have been attributed to differences in light quality, i.e., spectral composition, and greater temperature fluctuations in the field (Song et al. 2018). These factors may help explain why our study observed a strong effect of photoperiod, as natural conditions are often more complex and variable.

Several additional factors may influence the initiation of brood rearing, explaining the differences we observed between and within locations with regard to average temperature and day length. Food availability, for instance, could be significant, as the autumn cessation of brood rearing has been linked to a decline in pollen resources (Mattila and Otis 2007). While there is currently no direct evidence of the availability of resources as a cue, the importance of pollen on larvae and brood is well established (Nicolson 2011; Schmickl and Crailsheim 2001; van Dooremalen et al. 2013), suggesting it may influence brood initiation. Geographic factors, such as altitude and latitude, can be relevant in the timing of brood rearing as well. For example, lower altitudes and latitudes have been linked to earlier emergence dates in honeybees, likely due to photoperiod differences, temperature gradients, and other microclimatic conditions (Gordo et al. 2010). The absence of clear latitudinal trends in our results suggests that this effect may be masked or confounded by other factors. For instance, geographic differences between locations can also contribute to variations in honeybee ecotypes. In honeybees, ecotypes have adapted to match the specific environmental conditions (Le Conte and Navajas 2008). Genetic and environmental adaptations can influence phenological strategies (Edwards and Yang 2021); hence, colonies may regulate their brood development based on local conditions. Differences in heat tolerance and metabolism among honeybee ecotypes (Kovac et al. 2014) suggest that similar adaptations could influence brood rearing strategies in response to temperature. Furthermore, honeybees' internal clock, influenced by genetic background and local adaptations, may play a role in determining when brood rearing begins (Nürnberger et al. 2018). Finally, the differences we observed in phenological timing within locations may be due to genetic and physiological variation among individuals between colonies, influencing colony‐level interaction and responses (Jandt and Gordon 2016), such as brood rearing.

Our findings that both photoperiod and temperature influence the timing of brood‐rearing initiation in honeybees can have implications for the impact of climate change on honeybee populations. In plants, photoperiod and day length are the primary environmental cues for flowering (Andrés and Coupland 2012), which also seem to apply to pollinator plants like dandelion ( *Taraxacum officinale* ) (Templ et al. 2017), an important food source for honeybees in spring (Lowe et al. 2022). While our results show that honeybees rely on these same cues, the response of plants and pollinators to shared environmental cues may differ. Rising temperatures have been shown to advance both pollinator emergence and plant flowering times, but the rate of change can differ between pollinators and host plants, as well as across latitudes, potentially resulting in mismatches (Weaver and Mallinger 2022). Beyond geographical factors, these differences could be attributed to varying sensitivities to temperature (Olliff‐Yang and Mesler 2018). Such differential sensitivities could also suggest that the modulatory effect of photoperiod we found for honeybees may be different in plants, similar to how flowering dates vary among plant species with different photoperiod sensitivities (Zeng et al. 2024). Prolonged climate change could also most likely lead to asynchrony (Freimuth et al. 2022). Long‐term climatic shifts would likely require genetic changes across multiple generations to maintain synchrony with the environment (Le Conte and Navajas 2008). The capacity for such adaptations may be limited, as climate change‐induced range contractions could reduce the habitats available for honeybee colonies, with particularly severe declines projected in regions such as Africa and Europe (Rahimi and Jung 2024). If asynchrony occurs, mismatches between the initiation of honeybee brood rearing and plant flowering could harm colony food storage and exacerbate resource shortages, as brood rearing is estimated to consume half of a colony's annual honey stores (Debnam et al. 2024). Furthermore, honeybee colonies seem to have limited flexibility in adjusting brood rearing timing based on internal factors, such as food stores or parasite load (Ulgezen et al. 2024) which could further amplify the consequences of these phenological mismatches.

These findings highlight the importance of considering multiple environmental cues when predicting the effects of climate change on honeybees. Interacting species, such as plants and pollinators, may rely on shared cues but differ in their sensitivities, leading to shifts in phenology that occur at different rates. Such mismatches can disrupt interactions, with cascading effects on colony health, pollination services, and broader ecosystem resilience. Colonies may be especially vulnerable during the transition from late winter to early spring, when brood rearing begins but floral resources remain scarce or highly variable (Ulgezen et al. 2025). Management actions, such as increasing semi‐natural habitats within intensive agricultural landscapes (Verrier et al. 2024), may improve resource provisioning and help buffer against climate‐driven mismatches in resource availability. Accounting for shared environmental cues and how they may affect species differently can improve our ability to anticipate and mitigate climate‐driven disruptions to ecological interactions and vital ecosystem services such as pollination and help inform more effective ecosystem management strategies.

## Author Contributions

**Zeynep N. Ulgezen:** conceptualization (equal), formal analysis (lead), methodology (lead), visualization (lead), writing – original draft (lead), writing – review and editing (equal). **Coby van Dooremalen:** conceptualization (equal), writing – review and editing (equal). **Frank van Langevelde:** conceptualization (equal), methodology (supporting), visualization (supporting), writing – review and editing (equal).

## Conflicts of Interest

The authors declare no conflicts of interest.

## Supporting information


**Data S1:** ece372066‐sup‐0001‐Supinfo.docx.

## Data Availability

The data from honeybee colonies that support the findings of this study are openly accessible at https://beehealthdata.org. Ambient temperature data for locations were obtained from ECMWF Reanalysis 5, available at https://doi.org/10.24381/cds.adbb2d47.
