# Supplementary material for: Photoperiod and Temperature as Seasonal Cues for the Initiation of Brood Rearing in Honeybees
Source: Ecol Evol. 2025 Aug 27;15(9):e72066. doi: 10.1002/ece3.72066 (PMC12391022; doi:10.1002/ece3.72066)
Supplement: Supplementary file 1 — Data S1: ece372066‐sup‐0001‐Supinfo.docx. [file ECE3-15-e72066-s001.docx]

**Supplementary materials to:**

Photoperiod and Temperature as Seasonal Cues for the Initiation of Brood Rearing in Honeybees

Zeynep N. Ulgezen, Coby van Dooremalen, Frank van Langevelde^1^

In this supplementary material we included additional methods and results, supporting the main article. There is information on:

- Verification of day of brood initiation
- Relation between apiary latitudes and ambient temperature and photoperiod
- Model comparisons for Cox Proportional Hazards Model
- Comparison between weather data obtained from ERA5 and local station

*Brood Initiation*

We verified the initiation of brood rearing using multiple methods to ensure accuracy. The primary criterion was the first day of the year when in-hive temperature reached 33°C or higher, with brood rearing confirmed only if this temperature was maintained for at least three consecutive days (threshold method) (Villagomez et al., 2021). If these conditions were not met, the next occurrence was recorded as the start of brood rearing. However, since the distance between temperature sensor and brood cluster could possibly impact temperature recordings, causing errors or delays in detection, we used two additional verification methods. First, we analyzed graphical representations of in-hive temperature over time, using the data from sensors, identifying the first noticeable temperature increase in each colony as an indicator of the transition from a broodless state to brood rearing (graphical method) (Ulgezen et al., 2024). Second, in the B-GOOD project, checks of brood presence were conducted by opening the hive, every 21 days, recording the first observed brood occurrence (brood check method). We compared the day of the initiation of brood rearing identified by using the different methods with an ANOVA test, and Tukey’s HSD for post-hoc comparisons. The day of brood initiation detected by the various methods differed (F _2,171_= 17, p < 0.001). However, the main difference was between brood check method (mean = 90.5) and the other two methods: threshold method (p < 0.001) and graphical method (p < 0.001). Day of brood initiation was very similar between threshold method (mean = 65.2) and graphical method (mean = 65.7), and no significant difference was found between them (p = 0.9). Due to the lower frequency of the brood check method, this method was the least accurate of the three. As expected, the detection of brood initiation was delayed compared to the other two methods (Figure S1).


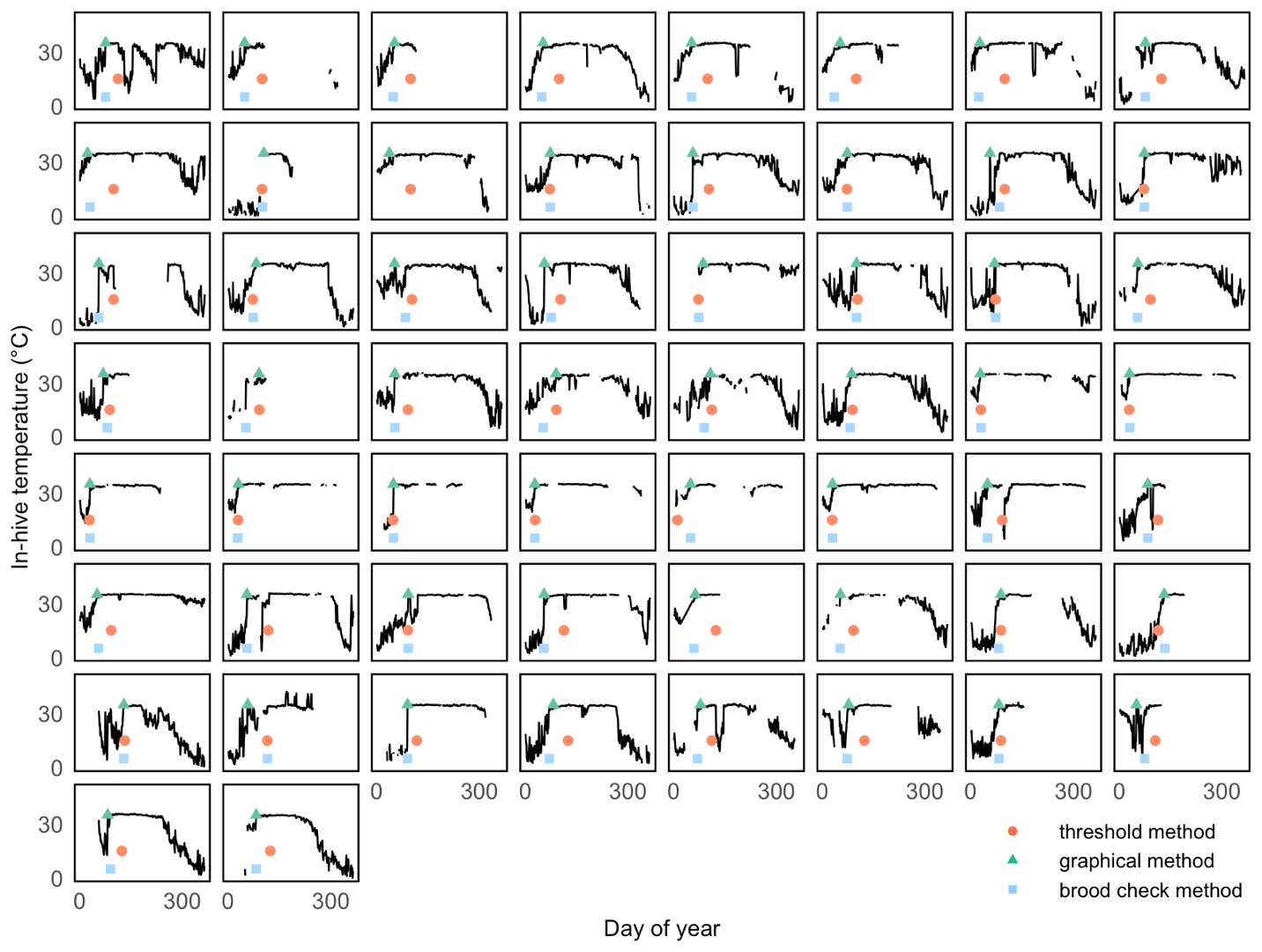


**Figure S1.** In-hive temperature for each colony over time. Lines represent daily in-hive temperature from sensors. Points indicate day of brood initiation as determined by each of three methods: (1) the threshold method (33°C maintained for ≥3 consecutive days), (2) the graphical method (visually identified temperature increase), and (3) the brood check method (first direct observation of brood presence during inspections).

*Temperature data*

**Table S1.** Relationship between temperature data obtained from local weather station and from ERA5 (Muñoz-Sabater et al., 2021) for each location. Analyses were done using a Pearson Correlation test.

| **country** | **R^2^** | **p-value** | **n** |
| --- | --- | --- | --- |
| BE | 0.81 | < 0.001 | 374 |
| CH | 0.99 | < 0.001 | 363 |
| UK | 0.99 | < 0.001 | 728 |
| DE | 0.99 | < 0.001 | 653 |
| FR | 0.99 | < 0.001 | 728 |
| PT | 0.99 | < 0.001 | 363 |
| RO | 0.99 | < 0.001 | 417 |
| NL | 0.99 | < 0.001 | 701 |

*Model Comparison*

**Table S2.** Model comparison for Cox Proportional Hazards Model used for determining the effect of ambient temperature and photoperiod on onset of brood rearing in honeybee colonies. To account for the potential lagged or cumulative effect of ambient temperature on brood initiation, we tested rolling mean temperature windows of 1-10 days. The table shows the AIC values for each model, and p-values for each temperature window. While models with higher smoothing windows (i.e. 8-10 days) yielded slightly lower AIC values, temperature was no longer significant predictor beyond 7-day window. This likely reflects signal loss due to over-smoothing, which diminishes meaningful short-term temperature variation. Therefore, the 7-day window was selected as the optimal balance between model fit and biological relevance.

| **window (days)** | **AIC** | **p-value** |
| --- | --- | --- |
| 1 | 37.87 | 0.007 |
| 2 | 45.39 | 0.001 |
| 3 | 42.49 | 0.001 |
| 4 | 35.07 | 0.002 |
| 5 | 32.96 | 0.004 |
| 6 | 30.86 | 0.005 |
| 7 | 28.21 | 0.008 |
| 8 | 18.80 | 0.054 |
| 9 | 15.63 | 0.107 |
| 10 | 12.67 | 0.196 |

*Apiary latitudes*


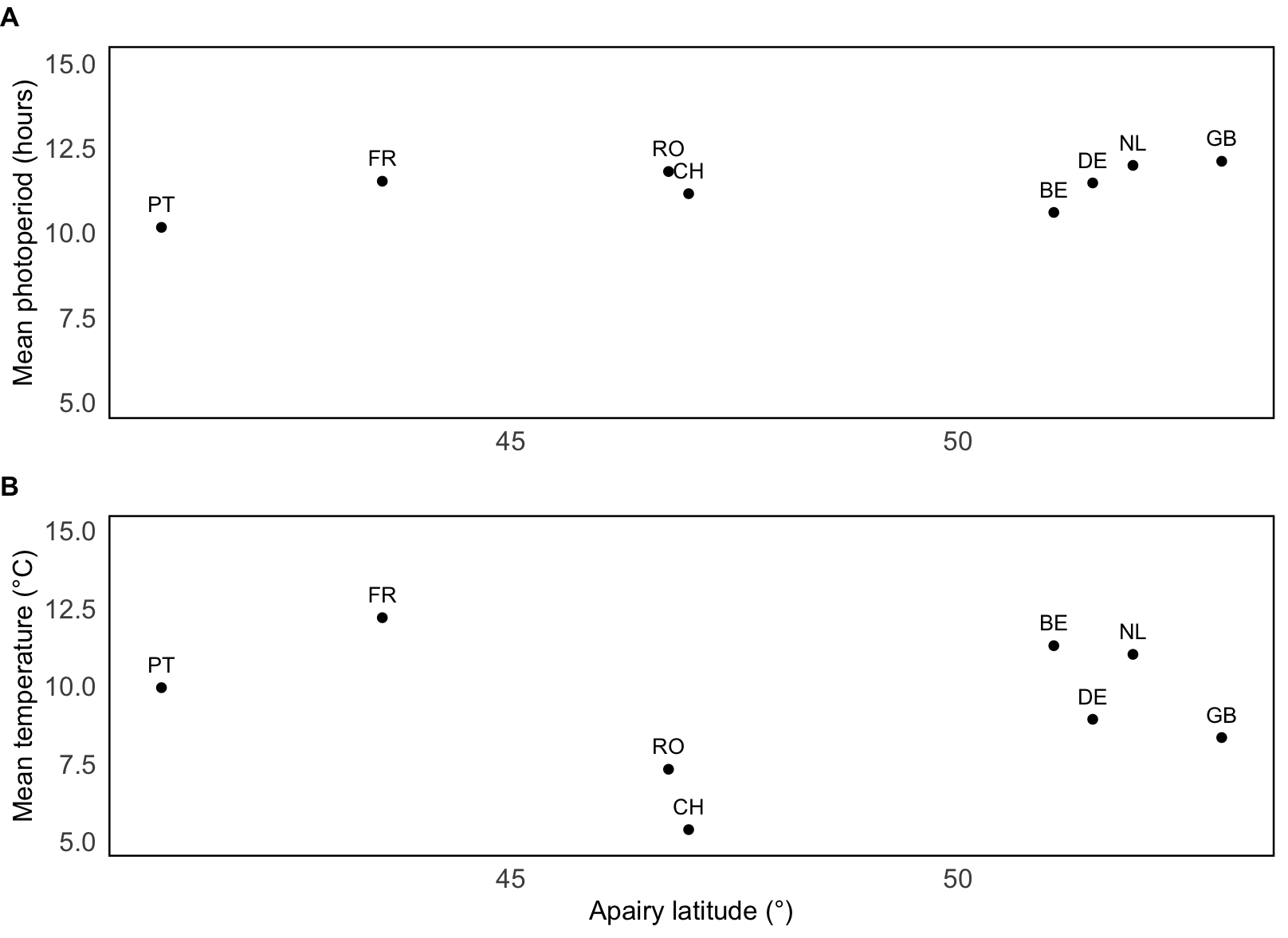


**Figure S2.** Relationship between each apiary latitude and (a) mean photoperiod, and (b) mean ambient temperature on day of brood initiation. Each point represents the average value for the country the apiary is located in, labeled accordingly. There is no visible pattern between latitude and either of these environmental factors.

**References**

Muñoz-Sabater, J., Dutra, E., Agustí-Panareda, A., Albergel, C., Arduini, G., Balsamo, G., . . . Hersbach, H. (2021). ERA5-Land: A state-of-the-art global reanalysis dataset for land applications. *Earth system science data, 13*(9), 4349-4383.

Ulgezen, Z. N., Van Langevelde, F., & van Dooremalen, C. (2024). Stress-induced loss of social resilience in honeybee colonies and its implications on fitness. *Proceedings of the Royal Society B, 291*(2014), 20232460.

Villagomez, G. N., Nürnberger, F., Requier, F., Schiele, S., & Steffan‐Dewenter, I. (2021). Effects of temperature and photoperiod on the seasonal timing of Western honey bee colonies and an early spring flowering plant. *Ecology and Evolution, 11*(12), 7834-7849.
